# Treatment strategies for amblyopia in adults: an overview of conventional methods and new technologies

**DOI:** 10.22336/rjo.2025.74

**Published:** 2025

**Authors:** Przemysław Ciszewski

**Affiliations:** Scientific Association of MedTech at the Center for Remote Learning and Educational Effects Analysis, Faculty of Medical Sciences in Katowice, Silesian Medical University, Katowice, Poland

**Keywords:** amblyopia treatment, adult amblyopia, adult neuroplasticity, dichoptic training, virtual reality therapy, a-tDCS = anodal transcranial direct current stimulation, AR = augmented reality, BCVA = best-corrected visual acuity, BDNF = brain-derived neurotrophic factor, cTBS = continuous theta burst stimulation, LTD = long-term depression, LTP = long-term potentiation, logMAR = logarithm of the minimum angle of resolution, tDCS = transcranial direct current stimulation, TMS = transcranial magnetic stimulation, tRNS = transcranial random noise stimulation, tRNS-R = transcranial random noise stimulation with reinforcement, V1 = primary visual cortex, VEP = visual evoked potentials, VR = virtual reality

## Abstract

**Aim:**

This review evaluates the efficacy of conventional and emerging therapies for amblyopia in adults, challenging the traditional notion of limited neuroplasticity beyond childhood. The study aims to compare the effectiveness of methods such as perceptual learning, dichoptic training, virtual reality (VR), and pharmacological interventions in improving visual acuity, binocular vision, and perceptual functions.

**Methods:**

A systematic literature search was conducted in PubMed, Google Scholar, Scopus, and Web of Science (1996–2025). Keywords included amblyopia treatment, adult neuroplasticity, dichoptic training, and VR therapy. Inclusion criteria prioritized clinical trials, systematic reviews, and meta-analyses focusing on adult amblyopia. Studies on pediatric populations, animal models, and non-scientific articles were excluded.

**Results:**

The reviewed studies indicated that conventional treatments, such as refractive correction and occlusion therapy, offer limited efficacy in adult amblyopia, with modest improvements in visual acuity. In contrast, emerging therapeutic strategies—including dichoptic training, virtual reality–based interventions, neuromodulation techniques, and selected pharmacological agents—demonstrated more substantial and sustained gains in both acuity and binocular function. These modern approaches appear to leverage neuroplasticity more effectively, particularly when combined with engaging, perceptually balanced stimuli and periodic reinforcement.

**Discussion:**

The reviewed evidence indicated that although conventional therapies offer limited improvement in adults, modern neuroplasticity-based methods provide more substantial and consistent benefits. Emerging techniques such as perceptual learning, dichoptic stimulation, and VR/AR interventions demonstrate significant gains not only in visual acuity but also in binocular integration. However, heterogeneous study designs and limited long-term data highlight the need for standardized protocols and further clinical validation.

**Conclusions:**

Adult amblyopia therapy is feasible due to retained neuroplasticity. Emerging technologies (VR, dichoptic training) and neuromodulation outperform conventional methods, particularly for binocular vision. However, individualized protocols and long-term efficacy studies are needed. Future research should optimize cost-effective, standardized treatments for clinical adoption.

## Introduction

### Characteristics of amblyopia

Amblyopia is a disorder of visual development resulting from a lack of adequate visual stimulation or abnormal processing of visual stimuli. Clinically, there are three primary forms: strabismic, refractive, and deprivation [**1**]. It is defined as a unilateral or bilateral reduction in best-corrected visual acuity (BCVA) without an identifiable organic cause. In unilateral amblyopia, the difference in visual acuity between the eyes is at least two lines on the Snellen or logMAR scale. In contrast, in bilateral BCVA, the vision in both eyes is below 6/12. Amblyopia is estimated to affect 1-6% of children, and up to 5.6% of adults often remain undiagnosed. Patients’ visual impairment is associated with slowed reading speed, impaired fine motor skills, and reduced stereoscopic vision. Due to the frequent lack of early diagnosis and treatment, visual impairment remains one of the leading causes of permanent visual impairment in the adult population as well. Clinically, three primary forms are distinguished: strabismic, refractive, and deprivation. The degree of amblyopia is also classified based on visual acuity: mild (BCVA 20/60 or better), moderate (20/60-20/100), and severe (20/100-20/400) [**2**].

### Purpose of the work

This study aims to evaluate the effectiveness of modern treatments for adult amblyopia and compare them with conventional methods. The review explicitly considers neuroplasticity-based therapies and technologies, such as perceptual training and virtual reality. The analysis aims to determine the effects of these methods on improving visual acuity, binocular vision, and perceptual function. The study is significant because it addresses a problem that was once considered highly limited in treatment options, if not impossible, in adults. Modern approaches offer new therapeutic options and hope for improving the quality of life of adult patients with amblyopia.

## Methods

To prepare this literature review, a systematic search for scientific articles was conducted in leading medical and scientific databases, including PubMed, Google Scholar, Scopus, and Web of Science. The temporal scope of the search included publications from 1996 to 2025, allowing us to include both classic and recent studies on the treatment of adult amblyopia. Emphasis was placed on describing the most recent scientific reports. Precise keywords and their combinations were used for the search, such as: “amblyopia treatment”, “adult amblyopia”, “neuroplasticity in amblyopia”, “perceptual training”, “virtual reality therapy”, “dichoptic therapy”, “transcranial stimulation”, and “pharmacological treatment of amblyopia”. Inclusion criteria included clinical trials, systematic reviews, and meta-analyses on the efficacy of both conventional and innovative therapies in adult patients with amblyopia. Papers dealing exclusively with the treatment of children, experimental studies on animal models, and non-scientific articles were excluded. The analysis of the collected data focused on evaluating the effectiveness of the therapies in improving visual acuity, binocular vision, and perceptual function, while accounting for the safety and feasibility of implementing the methods in clinical practice.

### Pathophysiology of amblyopia

Modern research indicates that amblyopia is primarily a cortical disorder, with central abnormalities in the primary visual cortex (V1). At that level, there is a reduction in the number of neurons responding to stimuli from the visually impaired eye, an increase in binaural suppression, and a loss of ability to process high spatial frequencies accurately. Disorders also extend to higher visual areas, leading to deficits in motion perception, contour integration, and other complex visual functions. Significantly, amblyopia also affects non-visual functions such as eye movement planning and attention processes, indicating its complex nature [**3**]. Visual functions mature during critical periods, often referred to as temporal windows of plasticity, during which sensory stimuli are essential for proper neural development. In humans, the most incredible sensitivity occurs during the first 18 months of life. Still, the visual system remains vulnerable until about 7-8 years of age, as evidenced by studies of binocular transfer in patients with early strabismus. During this time, unused connections between the eye and the brain can weaken or become completely inhibited, perpetuating visual impairment. For a long time, it was thought that treatment of amblyopia was only effective during this early period; however, more recent studies - both in animals and humans - have challenged this thesis, pointing to preserved, albeit limited, plasticity in adulthood as well [**4**].

### Neuroplasticity in adults

Research over the past four decades unequivocally confirms that the adult brain retains a significant capacity for structural and functional reorganization. Neuroplasticity processes include changes in neuronal morphology, such as stress-induced remodeling of dendrites; neurogenesis in the hippocampus and subcortical regions; and synaptic plasticity, including long-term synaptic strengthening and weakening (LTP/LTD). Neurogenesis in adult mammals, including primates, is modulated by environmental factors such as learning and hormones (cortisol, estrogen) and by molecular factors, such as brain-derived neurotrophic factor (BDNF) [**5**]. Contrary to previous beliefs, significant plasticity of the visual cortex also persists in adults, as confirmed by the recovery of visual function in the visually impaired eye after the loss of the dominant eye. This plasticity is based, among other things, on homeostatic mechanisms that can be stimulated by appropriate training or modification of visual stimuli. Although age influences the effectiveness of therapy, other factors - such as eye dominance, stress, or treatment environment - play an equally important role in the response to therapy. Thus, even in adults, there is potential for improved vision, especially when treatment accounts for individual patient characteristics and neuroplastic mechanisms [**6**]. Factors such as the balance between excitation and inhibition in neuronal networks have been shown to play a key role, and modulating them - for example, with neuromodulators or environmental stimulation - can increase the brain’s capacity for plasticity. Strategies such as perceptual learning or video games are promising, non-invasive methods for promoting neuroplasticity in adults with visual impairment [**4**].

### Conventional treatments for amblyopia in adults

### Refractive correction and occlusion of the dominant eye

A 2013 study by Fumiko Kishimoto and co-authors in adult patients aged 21-67 years showed that wearing full spectacle correction, combined with daily occlusion of the dominant eye for at least 1 hour, can lead to significant improvements in visual acuity in the visually impaired eye. The average improvement in distance vision was 2.4 logMAR lines, with 31% of participants achieving at least three lines of improvement. More favourable effects were observed in participants under 45 years of age and in patients with more severe baseline visual impairment. The treatment was well tolerated and its efficacy was maintained throughout the average 14-month follow-up period [**7**]. A study conducted in India between 2023 and 2024 evaluated the effect of occlusion therapy in 28 adult patients with anisometropic unilateral visual impairment. After 8 weeks of daily occlusion of the dominant eye for 3 hours a day, up to 93% of participants showed an improvement in visual acuity, with an average gain of 1.03 ± 0.94 logMAR lines. Treatment efficacy was the greatest in younger patients, with improvement in all patients in the 18-20 age group and only 50% among those over 24 years of age. The results indicate that a neuroplastic response to occlusion therapy is possible even in adults, although efficacy may decrease with age [**8**]. A study in 2023 showed that in adults with long-term visual impairment (mean age 33 years), a combination of 2 hours of daily occlusion of the healthy eye with visual training (30 minutes of the Amblyopia iNET programme and 1.5 hours of convergence exercises) for 12 weeks significantly improved visual acuity-by an average of 1.7 lines on logMAR arrays, with improvements in contrast sensitivity. Notably, the effects persisted after 24 weeks, even in patients who had previously tried other therapies without success. This suggests that shorter but systematic patching sessions combined with active visual training may be a viable treatment option for adult amblyopia [**9**].

### Atropine

Currently, there are few studies in adults using atropine drops to treat visual impairment. In studies of children, atropine has shown efficacy comparable to patching, and it is often better tolerated, less stressful for the child, and cosmetically acceptable. Despite these advantages, it remains a second-choice method in clinical practice, which may be due to the risk of systemic side effects such as dry mouth, photophobia, or headache [**10**]. One study compared atropine treatment to eye patching in patients after a “critical period” of vision development. The effectiveness of full-scale patching (covering the eye) was compared with atropine therapy (1% drops) in patients aged 8-20 years with anisometropic visual impairment. After 6 months, both groups achieved similar improvements in visual acuity (approximately 2.3 Snellen lines), although patching produced faster results (3.7 vs. 4.7 months). Although atropine caused more frequent ocular irritation, it was better accepted by patients than wearing a patch [**11**]. This study offers hope that this method will prove itself in adults.

### Modern technology and innovative treatment strategies

### Perceptual learning

A study by Levi and Polat, published in 1996, was among the first to dispel the myth that amblyopia is irreversible in adults. It showed that in adults with amblyopia, systematic training in the assessment of line shifts (Vernier acuity) can lead to significant improvements in visual function. Six participants performed precision visual tasks for several weeks, resulting in an average improvement of 46% in trained visual stimulus angle orientation, with effects partially transferring to the other eye (33%) but not to other visual tasks. Notably, one subject showed an impressive improvement in visual acuity from 20/80 to 20/22, challenging the previous belief that amblyopia is irreversible in adults and opening the door to therapies involving perceptual learning [**12**]. In a 2025 study, 10 days of contrast perception training in the visually impaired eye significantly improved contrast sensitivity (by an average of 25%) and restored binaural balance in adults with amblyopia. The training increased visual acuity in the trained eye by an average of 1.5 Snellen lines. The results suggest that even a brief intervention can modulate the plasticity of the visual system in adults, offering new possibilities for treating binocular vision disorders [**13**]. In contrast, another study showed that just 2.5 weeks of training with flickering stimuli (5 sessions of 30 minutes) significantly improved visual function in adults with amblyopia. Participants showed a 17% increase in temporal sensitivity (CFF), an average 0.12 logMAR increase in visual acuity, and improved stereopsis, as confirmed by VEP testing, which showed a 43% increase in visual response amplitude. Importantly, the effects spilled over into spatial function, even though the training focused on temporal aspects [**14**].

### Dichoptic training

In one study, eighteen visually impaired adults trained daily for 1 hour, for a fortnight, playing the game Tetris with special goggles. Half of the participants played monocularly, with their dominant eye covered, and the other half played dichoptically. The dichoptic-trained group achieved a significantly greater improvement in visual acuity (more than four times that of the monocular group), a marked improvement in spatial vision (stereopsis), and a significant reduction in interocular suppression. Significantly, when the monocular group was later transferred to dichoptic training, they also showed further considerable improvement. The effects persisted for 3 months, suggesting that abolishing suppression through dichoptic training may activate brain plasticity and be an effective treatment for adult visual impairment [**15**]. The authors of one study conducted an observational study on 20 adult patients with visual impairment who first underwent 40 hours of monocular training to improve visual acuity and contrast sensitivity. After this stage, 85% of the participants had improved visual acuity, but only 25% showed a slight improvement in stereopsis vision. This was followed by 20 hours of dichoptic training, which resulted in significant improvements in stereopsis and binaural integration in 60% of patients. This demonstrates that combining monocular and dichoptic training can increase the effectiveness of adult vision impairment therapy [**16**]. Dichoptic training appears to have an essential role in improving stereopsis in adults with visual impairment. One study evaluated the effectiveness of home-based dichoptic therapy using red-green filters in young adults with non-isometropic anisometropic visual impairment. After eight weeks of regular training, the study group showed a significant improvement in spatial visual acuity compared to the control group. The results indicate that this form of therapy may be a convenient and effective alternative to clinical treatment, especially in active patients [**17**].

### The use of virtual reality

Virtual reality (VR) technology has gained prominence in recent years as a state-of-the-art method to support the treatment of visual impairment, combining elements of perceptual learning and dichoptic stimulation. With its ability to influence neuronal activity and reorganise the cerebral cortex, VR can support improvements in functions such as visual acuity, contrast sensitivity, or stereoscopic vision [**18**]. One study evaluated the effectiveness of dichoptic training using VR goggles (Oculus Rift) in adults with anisometropic amblyopia. After eight 40-minute sessions with the therapeutic game “Diplopia Game”, a significant improvement in visual acuity was observed, with an average of 0.15 logMAR, and the percentage of patients achieving 20/40 or better acuity increased from 30% to 47%. There was also an improvement in stereopsis, with the number of patients with unmeasurable stereopsis falling from 47% to 12%. The results suggest that VR therapy may be effective in reducing interocular suppression and promoting binocular vision integration in adult patients with amblyopia [**19**]. A study evaluating the effectiveness of the VR game AmbliOK® in treating adult amblyopia showed mixed but promising results. The cases of four adult patients with anisometropic visual impairment were analysed. After four weeks of training (10 hours in total), all participants showed an improvement in visual acuity (0.2 to 1.0 logMAR on average), but in two of them the effect did not persist after one month. An increase in contrast sensitivity, especially at low spatial frequencies, and a marked improvement in stereopsis were also noted. The reduction in interocular suppression and the maintenance of positive effects even one year after therapy in one patient suggest that VR can be an effective tool in visual neurorehabilitation. However, effectiveness may depend on individual patient characteristics [**20**]. A pilot study conducted in 2023 evaluated the efficacy of anisometropic amblyopia therapy in adults using an inexpensive VR system based on anaglyph technology and a smartphone. The treatment lasted six months. Participants showed significant improvements in visual function: mean visual acuity in the visually impaired eye increased from 0.73 to 0.48 logMAR, and spatial vision and contrast sensitivity improved. An advantage of the system, apart from its effectiveness, was its accessibility and patients’ good cooperation with therapeutic recommendations. Although a limited response to treatment was observed in two patients, the results indicate the potential of this method as an affordable alternative to traditional therapies, especially in resource-limited settings [**21**].

### Transcranial stimulation

Non-invasive brain stimulation techniques, such as transcranial direct current stimulation (tDCS), can modulate brain activity and are promising for the treatment of visual impairment, mainly because they can be used at home. Anodal tDCS (a-tDCS) increases neuronal excitability, which can reduce inhibition in the visual cortex and support the effects of visual rehabilitation, especially after stroke. The study was conducted on a group of 16 young adults with visual impairment to evaluate the effectiveness of combining video game-based dichoptic therapy with anodal transcranial direct current stimulation of the visual cortex. Compared to dichoptic therapy alone, the combined treatment resulted in a greater improvement in binocular visual acuity (stereopsis). This demonstrates that additional stimulation of the visual cortex can increase the effectiveness of treatment for adult visual impairment [**22**]. Another variant of transcranial stimulation is magnetic stimulation (TMS). After transcranial theta-burst stimulation (tTBS) in visually impaired patients, visual acuity in the affected eye improved. In addition, a significant improvement in stereopsis was observed [**23**]. Five-day transcranial random noise stimulation (tRNS) of the visual cortex in adults with amblyopia improved contrast sensitivity and visual acuity briefly, with the effect peaking after the first session and gradually waning over time. After 28 days, only the improvement in visual acuity persisted [**24**]. In contrast, a recent pilot study showed that in adults with visual impairment, multiday transcranial random noise stimulation (tRNS) with monthly stimulation reinforcement (tRNS-R) maintains improvements in visual function, such as visual acuity, contrast sensitivity, and stereoacuity, for at least 6 months. In the group without regular reinforcement, the effects of improvement disappeared after only one month, indicating the critical role of continued therapy in achieving long-term benefits [**25**].

### Augmented reality technology

Augmented reality (AR) is a technology that combines the real world with digital, computer-generated information, enabling real-time interaction in three-dimensional space. Unlike virtual reality, AR does not isolate the user from the environment, but enriches it, for example, by overlaying visual or audio cues. In Milgram’s continuum, AR falls between reality and a completely virtual world, closer to the former [**26**]. A 2017 study used an augmented reality (AR) platform with an embedded contrast-balancing paradigm to enable daily, at-home binocular vision training in adults with visual impairment. After 6 weeks of use, there was a marked improvement in visual acuity and stereopsis in the majority of participants, along with a reduction in interocular suppression, which persisted for two more months after treatment [**27**]. A more recent study used AR to selectively enhance the parvocellular pathway, which is particularly impaired in patients with visual impairment. Training with flickering noise masking low spatial frequencies led to sustained improvements in visual acuity, high-frequency sensitivity, and improved dominance of the visually impaired eye [**28**].

### Pharmacological modulation of brain plasticity

### Modulation of the GABA system

In adults, brain plasticity is limited by, among other things, structural and functional inhibitory mechanisms such as periventricular networks or a disturbed balance of excitation and inhibition. Studies in animal models show that removing these barriers, either pharmacologically or via neuromodulation, can restore plasticity and improve visual function, opening up therapeutic possibilities in humans as well [**29**]. It has been shown that short-term visual deprivation of one eye in adults leads to a decrease in GABA levels in the visual cortex, which correlates with increased visual system plasticity. This implies that modulation of the GABAergic system may influence brain neuroplasticity. This gives hope for the use of GABA inhibitors in the treatment of visual impairment [**30**].

### Donepezil

In a pilot study involving people aged 9 to 37 years, daily donepezil use for 12 weeks resulted in an average improvement of 1.2 lines in visual acuity in the visually impaired eye, with 25% of participants improving by at least two lines. Notably, the effect persisted even after the end of therapy and was comparable in children and adults [**31**]. In contrast, another study found that donepezil did not enhance the effects of perceptual training in adults with visual impairment and may even slow learning and limit the transfer of acquired skills [**32**]. Donepezil may have potential in directly improving visual acuity in visually impaired adults, but its effect on perceptual rehabilitation is unclear and may be negative. Therefore, more extensive studies are needed to better define the efficacy and mechanisms of action of donepezil in the treatment of visual impairment.

### Fluoxetine

In a randomised trial of adults with visual impairment, fluoxetine, given for 3 months in combination with patching, significantly improved visual acuity compared with placebo. The improvement averaged 0.20 logMAR in the fluoxetine group compared to 0.08 logMAR in the control group. Furthermore, beneficial changes in VEP parameters were observed in fluoxetine-treated patients, supporting visual neuroplasticity [**33**]. Another study confirmed the efficacy of oral fluoxetine in treating visual impairment in patients aged 10 to 40 years. Administration of fluoxetine for 3 months, combined with patching of the better eye, resulted in significantly greater improvement in visual acuity than in the placebo group [**34**]. Another study evaluated the effect of fluoxetine (20 mg) combined with perceptual training and eye patching on improving visual acuity in 42 adult patients. The results showed significant improvements in vision in both the fluoxetine and placebo groups, suggesting that perceptual training rather than the drug itself played a key role. Although the study did not confirm fluoxetine’s efficacy, it did demonstrate the effectiveness of perceptual training in improving vision in adults with visual impairment [**35**].

### Levodopa with carbidopa

One study involving children and adults showed that three weeks of levodopa and carbidopa therapy, particularly at higher doses, led to improvements in visual acuity and contrast sensitivity in the visually impaired eye. However, these effects did not persist after treatment ended [**36**]. A case report describes a 46-year-old patient in whom 16 weeks of levodopa/carbidopa therapy led to a 2-line improvement in visual acuity in the visually impaired eye. The lasting effect persisted for at least six months. He is the oldest documented patient in whom such an improvement was observed [**37**].

### Cyticoline

Cytidine-5′-diphosphocholine (cytidine-5′-diphosphocholine), or CDP-choline (cytidine-5′-diphosphocholine), is a complex organic molecule that acts as an intermediary in the biosynthesis of cell membrane phospholipids. It increases brain metabolism and has been shown to raise levels of norepinephrine and dopamine in the central nervous system. Because of these neuro-modulatory properties, citicoline has been used to treat ocular conditions such as visual impairment (amblyopia) and glaucoma [**38**]. In a study of adults with visual impairment, a 15-day treatment with CDP-choline (cytidine-5′-diphosphocholine) markedly improved visual acuity, contrast sensitivity, and brain responses recorded during the VEP test. The improvement was evident in both eyes, although stronger in the visually impaired [**39**].

## Summary and conclusions

A review of current treatment strategies for adult amblyopia indicates that, despite the traditional belief in the visual system’s limited plasticity at this age, several promising therapeutic approaches can improve visual function. Conventional approaches, such as refractive correction and occlusion, remain the mainstay of therapy, but their effectiveness is limited and dependent on age and the severity of the visual impairment. In recent years, there has been a growing interest in modern methods based on neuroplasticity. Among the most promising are perceptual training, dichoptic therapy, and the use of virtual and augmented reality (VR/AR) technologies, which can improve visual acuity and restore stereoscopic vision by actively stimulating the visual system. Neuromodulatory techniques, such as transcranial direct current stimulation (tDCS) or transcranial magnetic stimulation (TMS), also show potential to enhance visual cortex plasticity, though they require further clinical research. Pharmacological approaches that modulate neurotransmission, including GABA inhibitors, selective serotonin reuptake inhibitors, and dopamine precursors, represent a fascinating but still experimental area of therapy, whose efficacy depends on individual patient characteristics and on their use in combination with other treatments. In conclusion, although progress is evident in the treatment of adult amblyopia, further clinical research is needed to optimise therapeutic protocols, identify predictors of treatment response, and develop personalised treatment strategies. It is also essential to standardise training methods and assess their availability and cost-effectiveness to enable wider implementation of modern therapies into clinical practice.
